# PlanText: Gradually Masked Guidance to Align Image Phenotypes with Trait Descriptions for Plant Disease Texts

**DOI:** 10.34133/plantphenomics.0272

**Published:** 2024-11-26

**Authors:** Kejun Zhao, Xingcai Wu, Yuanyuan Xiao, Sijun Jiang, Peijia Yu, Yazhou Wang, Qi Wang

**Affiliations:** ^1^ State Key Laboratory of Public Big Data, School of Computer Science and Technology, Guizhou University, Guiyang 550025, China.; ^2^ School of Information, Guizhou University of Finance and Economics, Guiyang 550025, China.

## Abstract

Plant diseases are a critical driver of the global food crisis. The integration of advanced artificial intelligence technologies can substantially enhance plant disease diagnostics. However, current methods for early and complex detection remain challenging. Employing multimodal technologies, akin to medical artificial intelligence diagnostics that combine diverse data types, may offer a more effective solution. Presently, the reliance on single-modal data predominates in plant disease research, which limits the scope for early and detailed diagnosis. Consequently, developing text modality generation techniques is essential for overcoming the limitations in plant disease recognition. To this end, we propose a method for aligning plant phenotypes with trait descriptions, which diagnoses text by progressively masking disease images. First, for training and validation, we annotate 5,728 disease phenotype images with expert diagnostic text and provide annotated text and trait labels for 210,000 disease images. Then, we propose a PhenoTrait text description model, which consists of global and heterogeneous feature encoders as well as switching-attention decoders, for accurate context-aware output. Next, to generate a more phenotypically appropriate description, we adopt 3 stages of embedding image features into semantic structures, which generate characterizations that preserve trait features. Finally, our experimental results show that our model outperforms several frontier models in multiple trait descriptions, including the larger models GPT-4 and GPT-4o. Our code and dataset are available at https://plantext.samlab.cn/.

## Introduction

Plant diseases are one of the key factors in crop yield reduction; hence, effective diagnosis and management tools are essential for food security. According to statistics [1], plant diseases reduce yields of crops such as wheat, rice, and corn by more than 17% and up to 30%. However, early detection of plant diseases is important to mitigate crop losses. Therefore, diagnosing plant diseases is essential for field management, and promoting computerized identification efficiently enables timely detection and diagnosis.

To address the plant disease diagnosis problem, deep learning-based methods have become a mainstream solution for cost-effective disease management, replacing traditional manual diagnosis. These methods achieve high precision and fast diagnosis by utilizing large-scale data to learn high-quality disease phenotypic features, thereby adapting to various types of diseases in different environments. In general, these deep learning-based phonemic methods provide effective research ideas for plant disease diagnosis in smart agriculture. Specifically, phenotype-based methods for the diagnosis and management of plant disease identification, detection, and segmentation all show great potential.

Visual networks, like convolutional neural networks (CNNs) [2–4], visual geometry group (VGG) [5,6], and transformers [7], are applied in the field of plant disease recognition. They also employ techniques such as transfer learning [8,9], deep feature fusion [10], attentional mechanisms [11], lightweight network design [12,13], and loss function optimization [14] to enhance model accuracy. In plant disease detection, researchers commonly utilize techniques such as Nuru [15], you only look once (YOLO) [16–21], faster region-based CNN [22], and DenseNet [23–25] for locating diseases accurately.

In the realm of plant disease segmentation, researchers utilize methods including automatic image segmentation for precise delineation [26,27], semantic segmentation to accurately identify disease regions [28], multiscale information processing to capture detailed features across different scales [29,30], and integrated learning frameworks aimed at comprehensive disease phenotype extraction from images [31,32]. Overall, these aforementioned recognition, detection, and segmentation studies can discriminate against crop diseases more accurately and locate them. However, due to the single source of data and simple usage scenarios, it is difficult for these methods to achieve effective plant disease diagnosis and management in real scenarios.

To address the single-modal problem, researchers explore the use of multimodal learning techniques to fuse information from a variety of sensors and data sources. These techniques are applied in medicine, human–computer interaction, smart agriculture, and large models.

**Fig. 1.**
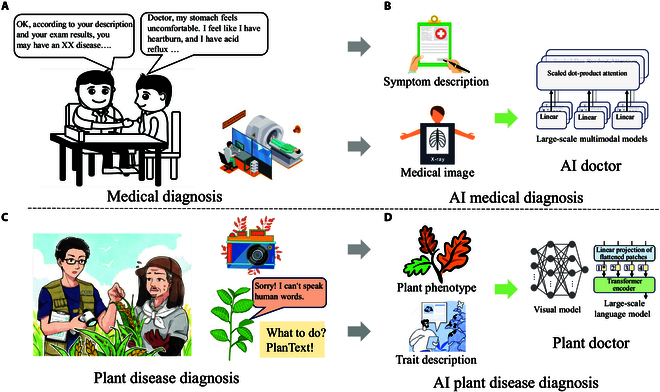
Comparison between medical and plant disease diagnoses by humans and artificial intelligence (AI). (A) A doctor diagnoses a patient based on the patient’s history. (B) Medical instruments perform detailed tests, with recorded data training multimodal doctor models. (C) Plants are diagnosed using cameras and our model (PlanText). (D) Traits and phenotypes are aligned to generate large-scale multimodal plant disease diagnostic models. These multimodal models, initially developed for human healthcare, are now extended to plant disease diagnosis through our PlanText model.

Although plant disease diagnosis shares similarities with medicine, the use of multimodal techniques is more effective and established in medicine than in plants. As shown in Fig. 1, in the medical field, data from patients’ self-reported illnesses, medical instrumentation, and doctors’ analyses of their illnesses form a medical multimodal medical database, which provides support for intelligent diagnosis and treatment. Moreover, multimodal technology [33–36] is used for learning these data to build artificial intelligence (AI) doctors [37,38] to assist doctors in all medical decisions and improve resource utilization. Overall, these multimodal methods show strong potential to handle complex datasets and cope with a wide range of environmental conditions, contributing to the efficiency and sustainability of production. In the field of botany, the application of text generation models primarily involves generating natural language descriptions, extracting key information from literature, and writing disease reports. Generative pretrained transformer (GPT) series [39–41] models can provide descriptions of plant characteristics and diagnostic methods, helping users understand plants. Additionally, models like bidirectional encoder representations from transformers (BERT) [42] are used for quickly extracting important information from botanical literature. The application of these models enhances the efficiency of information retrieval and dissemination in plant research and education.

However, compared with strong AI doctors, the whole plant field lacks text description methods, which hinders the application of multimodal technology in plant disease. Therefore, constructing a plant disease text description method is extremely necessary.

To this end, we propose a PlanText method to fill the gap, which can make the plant “speak” about its disease phenotype in this study. First, we collect 5,728 image phenotypic diagnostic reviews from 10 plant pathologists and hire 25 professionals in the field of plant diseases to annotate 21,000 new disease images with trait labels for PlanText model training and validation. Second, we propose a PhenoTrait text description model, which consists of a global and heterogeneous feature encoder and a switch attention decoder. The encoder extracts heterogeneous features and merges global features from disease images and description templates to provide a comprehensive representation of the data. The decoder uses heterogeneous features to dynamically balance attention between visual and textual information to generate detailed and accurate feature descriptions, ensuring accurate and context-aware output. Third, we design a 3-stage masking-guided method to align plant phenotypes and trait descriptions. More specifically, we reconstruct the corpus in the first stage, to acquire the structural features of the discourse for the optimization of model performance. In the second stage, the model generates the specified corpus through phenotypic descriptors (e.g., “color” and “texture”). In the final stage, the discourse descriptions are generated by aligning the model’s visual and textual feature extractors. The core semantic structure is preserved during the generation process through these 3 stages, while image features are embedded into the generated text. We design a phenotypic trait label extraction method by searching for the optimal feature labels in the corresponding trait library and evaluate the generated phenotype descriptions by calculating their similarity to the real labels, which helps in validating the accuracy and professionalism of the generated labels. Finally, the comprehensive experiments show that our model outperforms GPT-4 [43] as well as GPT-4o [44], in terms of both semantic preservation and integration of visual information. Our model promotes the intelligent development of plant disease identification and management, providing a new approach to botanical data augmentation.

The contributions of this work are summarized as follows:

• We propose the PlanText framework to improve the ability to generate detailed and accurate textual descriptions of plant disease phenotypes from images.
• We build a multimodal image–text database of 21,000 image–text pairs and 126,000 phenotypic labels for 63 plant species and 310 diseases to aid in plant disease diagnosis and management.
• We open-source contribute the PhenoTrait text description model that utilizes dynamic attention to generate accurate trait descriptions from disease images and text templates.
• We design an innovative validation method for image description phenotypic trait label extraction to assess the accuracy of generated descriptions based on the corresponding phenotypic features.

## Materials and Methods

In this section, we introduce gradually masked guidance to align plant phenotypes with trait descriptions for disease image diagnostic text method (PlanText) in detail. The “Dataset” section describes the dataset required for the experiment. The “Methods” section describes the framework of PlanText. The “Object function” section explains the objective function of PlanText with loss terms. The “Phenotypic trait label extraction” and “Evaluation indicator” sections report the details of the evaluation methods for the experiments.

### Dataset

Both the construction of plant disease images and the labeling of datasets are crucial for improving the accuracy and efficiency of disease diagnosis. This process provides a basis for training and evaluating deep learning models to improve early disease detection and complex disease diagnosis, thereby increasing the efficiency of agricultural production. Research on deep learning recognition of plant diseases based on datasets is a captivating topic. For instance, the PlantVillage Dataset [45] offers a collection of plant disease images with the aim of advancing research in plant disease recognition. The LifeCLEF [46] plant observation data encompass images, sounds, and textual data for biodiversity monitoring and species identification research. Inspired by the application of computerized multimodal macromodels to medicine, medical professionals can quickly obtain diagnostic results from symptoms and medical device intelligence reports. Similarly, plant disease diagnosis can be obtained in real time from multimodal modeling. However, current datasets lack key textual descriptions of plant phenotypes, which hinders the application of large multimodal models for real-time plant diagnosis. To address this challenge, we employ a generative model of plant disease trait descriptions, augmenting expert-provided statements to make them more comprehensive and applicable to a wider range of images. However, the current studies still suffer from the problem of insufficient model accuracy and data diversity. Therefore, we construct a comprehensive dataset that contains detailed plant disease images and labels to promote model training and enhance the accuracy and improve the reliability of disease diagnosis.

#### Data source

For pre-preparation, we collect a series of image datasets, which contain characteristics of plant pests and diseases. The image data comes from our PlantPAD [47], filtering images from authoritative pathology sources, open-source community contributions, and our accumulated field data. We also conduct in-depth studies of articles from authoritative pathology sources, providing detailed insights into trait characteristics such as color, tissue texture, and morphological changes.

#### Expert annotation

**Fig. 2.**
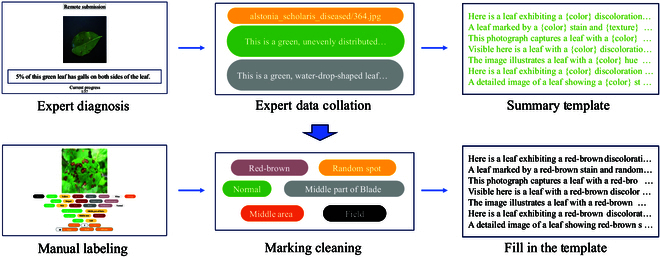
The 2 processes of dataset construction. Step 1: Two experts annotate diagnostic texts for the same image, extracting commonly used disease image description labels and generating expert-diagnosed text templates. Step 2: Employing crowdsourcing techniques, we manually label images and filled in labels into the templates, resulting in the creation of a comprehensive dataset.

Our labeling work is divided into 2 steps as shown in Fig. 2. As the first expert annotation, we consult 5 senior professors who are specializing in plant diseases, reference authoritative journals [48], and books [49,50], for carefully analyzing disease image data. With their guidance and advice, we construct high-standard plant disease annotation standards. The standards cover detailed features including color (black, green, yellow, brown, gray, red-brown, white), texture (spotted, striped, ring spot, netted spot, random spot), morphology (atrophy, wilt, rot, burn, perforation, normal), location (edge part of the blade, middle part of the blade), area (large area, middle area, small area), and address (field, lab). This comprehensive annotation not only enhances the specialization of the dataset but also improves the accuracy and the reliability of disease diagnosis. Meanwhile, in order to improve labeling efficiency and support multilanguage labeling, we develop a data annotation platform (the platform has built-in functions such as image display, text translation, and exporting of Word files; the code is at https://github.com/kej-shas/data-annotations). Finally, we invite 10 experts in the field to carefully analyze the images of plant diseases, and 2 experts simultaneously mark the text annotation of each plant disease image.

After 10 days, we obtain 5,728 texts from 2,894 images of plant diseases. By analyzing the text annotation features marked by the experts, we extract the image trait features and utterance structure marked by the experts, ensuring that all preconditions for manual annotation are met.

#### Human annotation

In the second stage of the manual annotation process, to further enrich the dataset, we develop our platform for characterizing disease images, which facilitates annotation through text templates summarizing expert annotation information. We invite 25 professionals in the field of plant diseases to analyze the images carefully with the expert annotation template information and annotate the specific characteristics of each disease in each image. After 1 month of meticulous work, we create 103,098 labels for 17,183 disease images, covering aspects such as color, texture, morphology, location, and area ratio. In this way, the accuracy and comprehensiveness of the dataset can then be ensured. Ultimately, we recombine these annotation tags into the original expert text descriptions, resulting in more detailed and specific image text descriptions.

**Fig. 3.**
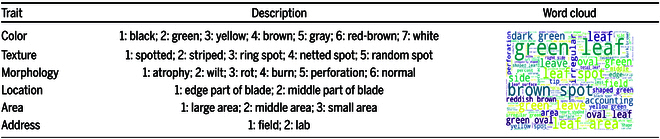
Comprehensive display of trait descriptions and word cloud.

The data collection and annotation efforts result in a comprehensive dataset of 17,183 plant disease images containing 103,098 labels and 5,728 texts. As shown in Fig 3, the dataset contains a variety of disease features including color, texture, morphology, location, and area ratio, ensuring a rich resource for training and evaluating deep learning models for plant disease diagnosis.

### Methods

#### PlanText framework

**Fig. 4.**
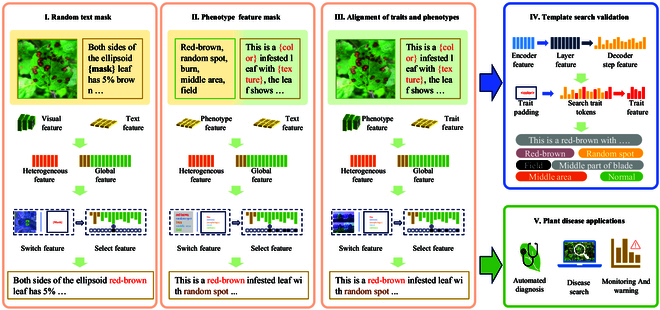
The proposed PlanText framework, which includes 3 components: a PhenoTrait text description model, a 3-stage gradual masking guide, and phenotypic trait label extraction. The PhenoTrait model extracts features from disease images and descriptions with encoders and a switch attention decoder. The stepwise masking technique aligns phenotypes and traits by revealing concealed text, ensuring accurate descriptions. The template search method improves model evaluation and labeling control. PlanText is used for disease recognition, health monitoring, alerts, and crop management.

To obtain consistent generation of plant disease image phenotypes and trait descriptions, we propose the PlanText framework, as Fig. 4 shows. First, the phenotype and the trait description template text are put into the PhenoTrait text encoding model for comprehensive and heterogeneous feature characterization. Second, the encoded features are fed into the decoder, which utilizes heterogeneous features and history constructs along with attention maps to generate detailed trait descriptions. Then, these descriptions are produced by dynamically optimizing the attention between image features and textual traits. In addition, we design a 3-stage mask bootstrapping strategy for training. Specifically, in the first step, disease phenotype images and text with masks are applied to reconstruct the proper names of feature descriptions and image phenotype traits. In the second step, we input phenotype labels with instructions and feature description templates to filter relevant features in the disease phenotype images according to the instructions. In the third step, the features of the disease phenotype images are aligned with the phenotype labels from the second step to complete the alignment of the disease phenotype images with the phenotype label features. In the inference stage, we design a method to extract phenotypic trait labels. We employ a special token to populate trait text and generate a text template. This template is then used to search for trait description labels in the feature library, following the predicted sequence generated by the model. The similarity between the searched labels and the actual labels is calculated to measure the quality of the generated trait text. This approach ensures accurate metrics for evaluating the model. In addition, the template search allows us to control the labeling of all desired features, thus improving the applicability of the model.

#### Global and heterogeneous feature encoder

To extract the trait features of disease phenotypes for description generation, we design global and heterogeneous feature encoders. The main purpose of these encoders is to extract global disease image and description template latent variable information from the disease image and description text template feature extractors. Heterogeneous feature extraction is also implemented to capture the cross-features between the image and text and enhance the decoder’s cross-focus on the difference features of the image and graphic text.

**Fig. 5.**
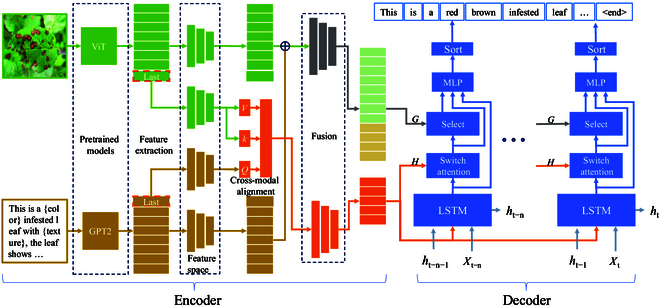
The details of PhenoTrait text description model. The left is the global and heterogeneous feature encoder, and the other side is the switch attention decoder.

Specifically, in the encoder module, as shown in Fig. 5, we extract disease image features $V_{i}$ and description template features using pretrained image (Vision Transformer [ViT] [51]) and text (GPT-2 [40]) models, respectively. For disease image feature processing, we use a linear transformation $W_{i}$ to map the features, which are extracted by disease image features, to a hidden space. The details are as follows:

$$V_{i}^{\prime }=\text{ReLU}\left(W_{i}\cdot V_{i}\right),$$ (1)

where *ReLU* represents the rectified linear unit for nonlinearity and $V_{i}$ is the output of the ViT [51]. Similarly, for the description template feature processing, we map the features to the same hidden space through linear transformation:

$$T_{i}^{\prime }=\text{ReLU}\left(W_{t}\cdot T_{i}\right),$$ (2)

where *ReLU* represents the rectified linear unit for introducing nonlinearity and $T_{i}$ is the output of the GPT-2 [40].

Next, we merge the disease image features and the description template features. After the linear transformation, we obtain the corresponding $V_{s}$ and $T_{s}$. These features are concatenated and further processed with nonlinear activation to obtain the final global feature *G*:

$$V_{s}=W_{a}\cdot V_{i}^{\prime },\;T_{s}=W_{b}\cdot T_{t}^{\prime },$$ (3)

$$G=\text{ReLU}\left(W_{\mathrm{ac}}\cdot \text{ReLU}\left(W_{\mathrm{ab}}\cdot V_{s}+W_{\mathrm{ab}}T_{s}\right)\right),$$ (4)

where $W_{a}$, $W_{b}$, $W_{\mathrm{ab}}$, and $W_{\mathrm{ac}}$ are the learnable weight matrices of the model for feature transformation and fusion.

Furthermore, these features are fused by tandem operations and further nonlinear activations to obtain overall strong features. After summarizing the disease crossover features, we propose a multiple attention mechanism to align the description template features with the disease image features. After obtaining the aligned features, we project the concatenated features through linear transformation $W_{p}$ to obtain the final heterogeneous features:

$$\text{aligned}\;\_\;\text{features}=\text{MultiheadAttention}\left(G_{\text{last}},V_{\text{last}},V_{\text{last}}\right),$$ (5)

$$\text{heterogeneous}=W_{p}\;\cdot \;\text{concatenate}\left(\text{aligned}\_\text{features},G_{\text{last}}\right),$$ (6)

where *MultiheadAttention* refers to the multihead attention mechanism, $G_{\text{last}}$ is the output feature of the last layer of GPT-2 [40], and $V_{\text{last}}$ is the output feature of the last layer of the ViT [51]. The 2 $V_{\text{last}}$ are the same to simultaneously use the same visual features in the multihead attention mechanism, enhancing the capture of relationships between images and text, originating from the feature maps of the image or the output of the encoder, and $W_{p}$ is a parameter matrix used to map the spliced features to a new feature space.

Overall, we focus on obtaining both global and heterogeneous features through feature alignment and fusion techniques. We also reduce the complexity of description template generation by focusing in depth on image feature generation through text-guided modeling phenotype and trait alignment. External text and heterogeneous disease image features are also proposed to divide the trait text description task in the decoder into professional text generation and disease image feature description selection.

#### Switch attention decoder

**Fig. 6.**
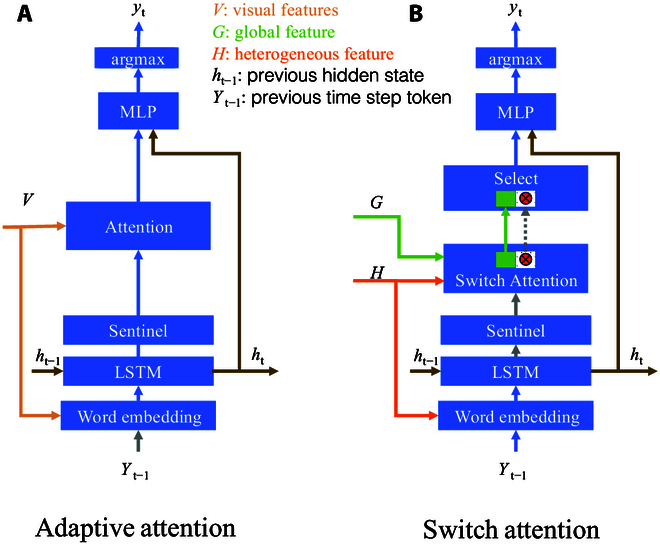
Comparison of different attentional decoders. (A) The adaptive attention [52] model is used to select important features dynamically among the given image region features to enhance the accuracy of description generation. (B) Our switch attention decoder further enhances multimodal feature fusion by introducing a switching mechanism that dynamically balances between image and text contexts to generate more accurate and context-aware descriptions.

In the phenotypic trait alignment task, integrating heterogeneous image and text features poses challenges, particularly due to the long-distance dependencies between subtle image features and textual descriptions. Traditional attention mechanisms struggle to dynamically balance these contexts, often resulting in inaccurate descriptions. As shown in Fig. 6, the conventional model for describing image features incorporates adaptive attention [52], long short-term memory (LSTM), image attention, and residual networks to generate textual descriptions step by step. However, when the text becomes overly complex, there is a tendency to prioritize fitting the text features at the expense of capturing the image phenotype features. To address this problem and facilitate dynamic switching between image and text contexts, we propose the switch attention decoder. This mechanism incorporates switching to effectively balance multimodal information, enhancing the accuracy and context awareness of disease descriptions.

To convert the words and images in the text into dense vector representations, we obtain word embedding vectors from a pretrained word vector model and the mixture of heterogeneous features (*H* in Fig. 6B). At each time step *t*, the current word embedding vector is concatenated with the global image–text fusion feature *fused* to form the input $x_{t}$ for the current step. This step aims to combine contextual information with image–text information.

We employ LSTM networks [53] for decoding. These networks operate by taking the current time step’s input $x_{t}$ along with the previous time step’s state information $h_{t-1}$. The LSTM then produces the current time step’s $h_{t}$ (hidden state) and updates *states* (cell state) accordingly.

$$h_{t},\text{states}=\text{LSTM}\left(x_{t},\text{states}\right).$$ (7)

In order to enhance the model’s ability to capture long-distance dependencies in text, the assisted attention mechanism and the selection block need to determine when to focus on which parts of the disease image. The model introduces a gating mechanism, where ${\text{gate}}_{t}$ is obtained through linear transformation and the sigmoid activation function. The gate is used to determine whether to retain information from the current cell state, generating the sentinel vector $s_{t}$. The vector can be used in subsequent attention mechanisms to help decide when the model should focus on which parts of the disease image, and the details are as follows:

$${\text{gate}}_{t}=\sigma \left({\text{affine}}_{x}\left(\text{dropout}\left(x_{t}\right)\right)+{\text{affine}}_{h}\left(\text{dropout}\left(h_{t-1}\right)\right)\right),$$ (8)

$$s_{t}={\text{gate}}_{t}\times \text{tanh}\left({\text{cell}}_{t}\right).$$ (9)

To achieve dynamic regulation of attention allocation, enhance the fusion of heterogeneous data such as image and text contexts and enhance alignment between phenotypes and traits. As shown in Fig. 6, the switchable attention mechanism enhances traditional attention by introducing a switch value that dynamically controls the balance between image and text contexts. This mechanism reaches balance using computing attention scores between query and key vectors, scaling them, and applying a sigmoid activation to obtain attention weights. These weights are modulated by the switch value, enabling the model to selectively attend to relevant information from both modalities. This mechanism facilitates the effective fusion of multimodal features, enabling more robust and context-aware caption generation in complex tasks like image understanding and description. The details are as follows:

$$\alpha =\text{sigmoid}\left(\frac{Q^{\prime }\cdot {K^{\prime }}^{T}}{\sqrt{\text{key}\_\text{size}}}\right),$$ (10)

$$S=\text{sigmoid}\left(W_{s}\left(\alpha \right)\right),$$ (11)

$$S_{\mathrm{hat}}=S⊙V_{I}^{\prime }+\left(1-S\right)⊙V_{T}^{\prime }.$$ (12)

where $Q^{\prime }$ and $K^{\prime }$ represent linearly transformed query and key vectors, respectively. $W_{s}$ is the linear layer for the switch value computation, and ⊙ denotes element-wise multiplication. $V_{I}^{\prime }$ and $V_{T}^{\prime }$ are the visual and textual features split by the global feature *G* from the encoder layer, respectively.

In order to integrate multiple attention mechanisms and modules, the generated outputs are optimized to enhance the performance and effectiveness of the model in complex multimodal tasks. The selection block that we propose in our model combines various attentional mechanisms, including a sentinel mechanism for capturing distant dependencies, switchable attention for dynamically balancing image and text contexts, and image spatial attention for focusing on salient visual features. These components are integrated to generate final scores for output captions, allowing the model to effectively leverage multimodal information and produce accurate and context-aware captions in diverse scenarios. The specific expression is as follows:

$$z_{t}=W_{h}\cdot \text{tanh}\left(W_{v}\cdot S_{\mathrm{hat}}+W_{g}\cdot h_{t}⊙1^{T}\right),$$ (13)

$$z_{t}^{\mathrm{ext}}=w_{h}\cdot \text{tanh}\left(W_{s}\cdot s_{t}+W_{g}\cdot h_{t}\right),$$ (14)

$$\alpha_{t}^{\mathrm{ext}}=\text{softmax}\left(\left[z_{t},z_{t}^{\mathrm{ext}}\right]\right),$$ (15)

$$\beta_{t}=\alpha_{t}^{\mathrm{ext}}\left[:,:,-1\right],$$ (16)

$$\alpha_{t}=\text{softmax}\left(z_{t}\right),$$ (17)

$$C_{\mathrm{hat}}=\beta_{t}\cdot s_{t}+\left(1-\beta_{t}\right)\cdot \alpha_{t}\cdot S_{\mathrm{hat}},$$ (18)

$$\text{scores}=\mathrm{mlp}\left(\text{dropout}\left(S_{\mathrm{hat}}+C_{\mathrm{hat}}+\text{hiddens}\right)\right),$$ (19)

where ⊙ denotes element-wise multiplication and [:,:,−1] selects the last column of $\alpha_{t}^{\mathrm{ext}}$ to obtain $\beta_{t}$. $W_{v}$, $W_{g}$, and $W_{s}$ are the linear layers. $S_{\mathrm{hat}}$ denotes visual and text switches, $h_{t}$ denotes LSTM hidden values, and $s_{t}$ is the sentinel module feature.

#### Gradually masked guidance

**Fig. 7.**
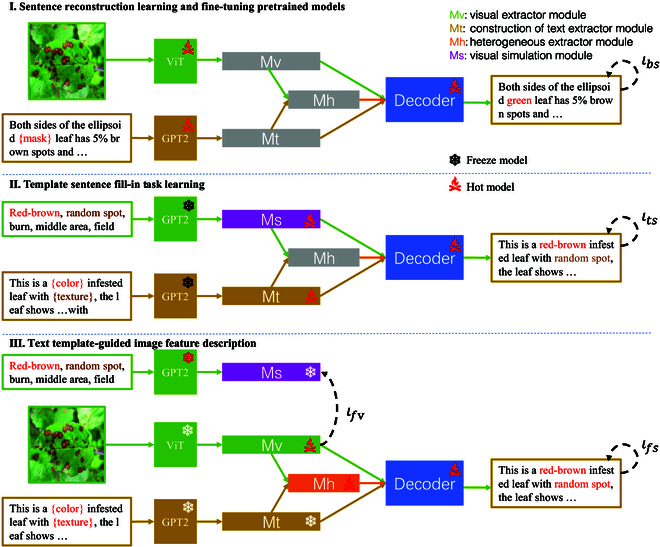
The PlanText gradually masked guidance. 1) Random text masking, used for training the basic image–text integration model. 2) Image feature masking, replacing phenotypic features of images with trait labels to enhance textual descriptions. 3) Text instruction-level masking, precisely aligning image features with text descriptions to generate accurate descriptions of plant diseases. This strategy utilizes progressive masking and attention-switching mechanisms to improve diagnostic capabilities.

In the field of botany and computer vision, there is a demand for accurate descriptions of plant diseases. Traditional image description models compute losses in text during the decoding stage and focus on text generation to maximize scores, leading to a substantial reduction in attention to image features. This limitation hinders practical applications such as accurate disease diagnosis in plant images with limited datasets or accurate description of plant diseases based on visual features. As shown in Fig. 7, we adopt the PlanText framework with a 3-stage strategy: a simplified visual extractor module (Mv), a text extractor module (Mt), and a heterogeneous extractor module (Mh). We introduce a gradually masked guidance mechanism and leverage multimodal characteristics. The process primarily involves 3 key steps:

1. Random text mask. This is done in order to quickly acquire the basic ability for reconstructing image–text features and generate text. We replace some of the trait feature words in the expert-labeled trait description text with the character “{mask}”. The model performs sentences that are repaired and reconstructed by disease image features with switch attention switch-related disease image phenotypes and utterance templates.
  By incorporating this masking strategy, we enhance the model’s capability to generate accurate descriptions from images, which in turn enables the final model to effectively shift its attention among various phenotypic features, leading to improved textual descriptions that align closely with the underlying visual information.
2. Image feature mask. After the previous step, we train the model to learn instructions that encompass both comprehensive and specific phenotype and trait label alignment features. Specifically, in GPT-2, the input phenotype label text simulates visual features (Ms), which simplifies the model’s task of learning commands to enhance alignment between image phenotypes and trait description labels. We replace “{mask}” in the description template section with feature description commands (such as “{color}”, “{texture}”, and “{morphology}”) to reconstruct the original sentence based on the phenotypic labeling. The forced text extraction module (Mt) learns the features of the sentence template. We train the switch attention module to turn off textual description templates when encountering trait description commands.
  This stage enhances the model’s overall capability by increasing dynamic attention switching between images and text. It also improves the alignment of phenotypes and traits, contributing to more accurate and relevant descriptions in plant disease identification.
3. Text instruction-level mask. In the final stage, we achieve precise alignment of image and text features to enable the model to accomplish the final task of trait-accurate description. We freeze the Mt module and extract image features using ViT [51]. The visual feature extraction module Mv is responsible for mapping the image features to the original text feature extraction. Meanwhile, the Mf module integrates the features extracted by the Mv and Mt modules. In this learning stage, we use the visual extraction Mv module to align outputs. Initially, attention focuses on activating image features to align with phenotypic features from images and descriptive text labels. When attention shifts to textual description features, the Mt module preserves core semantic structures.
  This phase substantially enhances the model’s ability to dynamically switch between image and text, resulting in tightly aligned descriptions that reflect expert knowledge and terminology.

In summary, key text features are extracted from plant disease sentence templates using level-by-level mask guidance to reconstruct discourse and access expert logic and domain-specific terminology. Extracting trait feature text from plant disease images ensures that the generated descriptions are tightly aligned with labeled instructions. The utility and reliability of the model are progressively improved.

### Objective function

In the first step of feature learning, our objective is to utilize image features for sentence restoration and reconstruction based on expert-annotated text inputs, aiming to rapidly acquire fundamental abilities in integrating textual and visual features and generating text. We can express the objective function of this step as minimizing the cross-entropy loss, where $l_{\mathrm{bs}}$ represents the cross-entropy loss:

$${\text{Object}}_{1}=\text{min}l_{\mathrm{bs}},\;l_{\mathrm{bs}}=-\sum_{i}y_{i}\text{log}\left({\hat{y}}_{i}\right).$$ (20)

In the second step of image feature learning, our objective is to train the model to fill in missing parts of the original sentence based on features, compelling the Mt module to learn the features of sentence templates to generate more realistic sentences. The loss function in this step is the same as in the first step:

$${\text{Object}}_{2}=\text{min}l_{\mathrm{ts}},\;l_{\mathrm{ts}}=-\sum_{i}y_{i}\text{log}\left({\hat{y}}_{i}\right).$$ (21)

In the final step, our objective is to align the features of text and image to enable the model to successfully accomplish the final description task. We adopt the cross-entropy loss $l_{\mathrm{fs}}$ as the objective function to validate the generated results and introduce the central moment discrepancy (CMD) loss $l_{\mathrm{fv}}$ to maximize the difference in image feature extraction. The loss function can be expressed as

$$l_{\mathrm{fs}}=-\sum_{i}y_{i}\text{log}\left({\hat{y}}_{i}\right),$$ (22)

$$l_{\mathrm{fv}}=D_{\text{cmd}}\left(y,\hat{y}\right)=\frac{1}{N}\sum_{i}^{N}{\left\|y_{\text{new}}-y_{\text{old}}\right\|}_{2},$$ (23)

where *N* is the number of samples, ${\left\|\cdot \right\|}_{2}$ represents the L2 norm (Euclidean distance). $y_{\mathrm{new}}$ is the output of the Mv module, and $y_{\text{old}}$ is the output of the Mv module in the second step.

By concatenating the values of the 2 loss functions, we introduce 2 hyperparameters, α and β. The final objective function can be represented as

$${\text{Object}}_{3}=\alpha \times \text{min}l_{\mathrm{fs}}+\beta \times \text{min}l_{\mathrm{fv}}.$$ (24)

The loss functions of each stage are similar in form, such as the cross-entropy losses $l_{\mathrm{bs}}$, $l_{\mathrm{ts}}$, and $l_{\mathrm{fs}}$, which allows them to be combined during optimization. To maintain the values of different loss functions on the same order of magnitude, we perform normalization. In the third stage, we eliminate the difference between the CMD loss $l_{\mathrm{fv}}$ and the cross-entropy loss $l_{\mathrm{fs}}$ by introducing the hyperparameters α and β. Thus, the final objective function can be expressed as

$$\text{Object}={\text{Object}}_{1}+{\text{Object}}_{2}+{\text{Object}}_{3}.$$ (25)

### Phenotypic trait label extraction

To improve the effectiveness and accuracy of text generation in all image captioning tasks, we design a phenotypic trait label extraction method, which is a template search inference method. First, we define a series of templates; each one specifies the desired structures and requirements for the generated text. During the text generation process, these templates guide the model to produce text that adheres to their specifications. Finally, we refine and validate the generated phenotypic feature labels to ensure that the output texts accurately reflect the structures and the requirements specified by the template.

#### Template search

**Fig.8.**
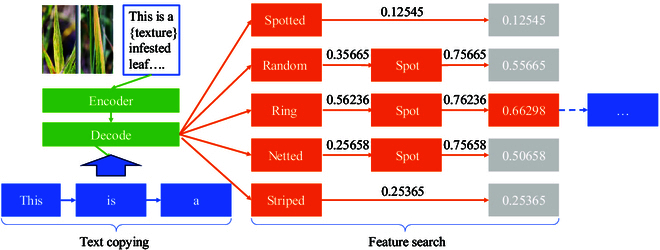
The phenotypic trait label extraction diagram, incorporating trait label search. It maps images to feature vectors, dynamically updates captions through template traversal, and efficiently searches for trait labels to refine results.

Template search prediction algorithms are used to search for specific disease trait texts from generic descriptors (e.g., “color”) from a feature lexicon. As depicted in Fig. 8, first, the input image is converted into a feature vector by an encoder. Then, the algorithm makes a deep copy of the description template texts and initializes relevant variables, including descriptor tokens for text traversal and a library of cached feature descriptions. These descriptor token dictionaries convert descriptors to their corresponding token values (e.g., “color” is indexed at 256; i.e., it is the 256th position in the sequence of the thermally encoded-word base, where token = 256).

#### Label extracting

Label extracting is applied to sort the scores of the labels in the thesaurus searched from the trait thesaurus to get the best trait description label. The algorithm progressively traverses each character of the template text, with the current character as $t_{i}$, looking for a specific descriptor token. When the descriptor token $C_{i}$ is found, the current caption sequence is updated, i.e.,

$$\text{captio}n^{\prime }=\left\{\text{caption},t_{\text{last}},\ldots ,t_{i-1}\right\}.$$ (26)

$t_{\text{last}}$ is the starting position of the last traversal to the descriptor tokens in the text template. Then, the disease features extracted by the encoder and the current caption are inputted into the decoder to generate the word bank weights for the next step. According to the decoder model, the dimensions of the results are adjusted and the results are token-sorted.

#### Template filling and costing

Template filling and costing generates completed utterances by filling tags into the description and quantifies the cost that the model needs to acquire each tag acquisition for the utterance combined with the image features to get the best result for each model. During the trait key search, the algorithm traverses all the trait labels of the descriptor feature description library and searches for these labels in the sorted results. After a successful match, the current key value is updated and its index value is recorded to calculate the search cost (i.e., how many traversals are needed to find the current trait label). For example, when encountering the “color” descriptor, the method traverses its feature library to find the red, yellow, brown, etc., trait labels and searches for these labels in the output. The number of traversals that is required to find these labels is recorded as the cost, along with the cost and the weight of the current label.

#### Efficient caching mechanism

To improve the efficiency of the algorithm, we design a cache logic processing to optimize computation and storage. After updating each caption, we traverse to the same descriptor character to retrieve the cached results of generated trait labels. The caching mechanism implements an efficient system to store computed results and speed up computation. For example, generated disease feature labels are cached to avoid repeated calculations of the same descriptors, thus improving the algorithm’s operational speed.

Refer to Algorithm 1 for the specific algorithm. In simple terms, we first initialize an empty text sequence *C* and then iterate through the words of the input text *X* one by one. Whenever we encounter the start marker of a paragraph, we extract the words from the previous marker or text to the current position, forming a new paragraph $P_{i}$. We use the model to generate text for this paragraph, then fill in the keywords as needed, and finally add the complete paragraph to the sequence *C*. We repeat this process until the end of the text, obtaining a complete text sequence *C* containing all the filled content ultimately.

### Evaluation indicator

Traditional automated evaluation metrics (e.g., Bilingual Evaluation Understudy [BLEU] [54], Recall-Oriented Understudy for Gisting Evaluation [ROUGE] [55], and Metric for Evaluation of Translation with Explicit Ordering [METEOR] [56]) are mainly used for evaluating machine translation and summary generation of plain text. These metrics cannot accurately judge image description generation tasks because they ignore the importance of image content to the description text, especially the lack of image description validation in the case of, e.g., plant disease trait descriptions and abstract descriptions. To address this problem, we have innovatively devised a technique called the phenotypic trait label extraction verification method. The method employs a phenotypic trait label extraction method that guides the model in generating the specific feature description labels we need, allowing us to evaluate the generated results more accurately.


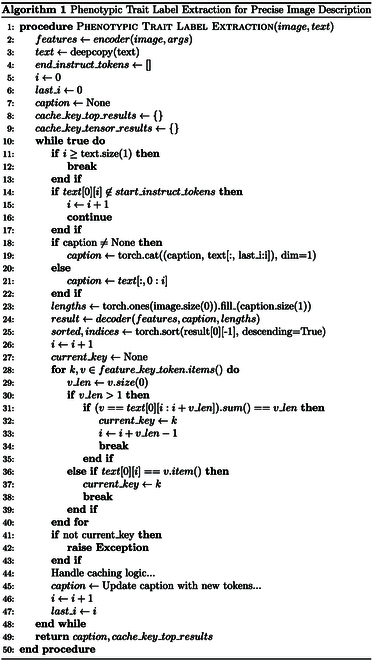


We adopt mathematical formulas to quantify the validation process. Suppose we have a set of disease feature labels $L=\left\{l_{1},l_{2},\ldots ,l_{n}\right\}$. Meanwhile, we have a database containing known disease features, denoted as $D=\left\{d_{1},d_{2},\ldots ,d_{m}\right\}$, where *m* is the number of disease features in the database. We define a validation function $V\left(l_{i}\right)$, which is used to determine whether the feature label $l_{i}$ is accurate:

$$V\left(l_{i}\right)=\frac{1}{m}\sum_{j=1}^{m}\mathrm{sim}\left(l_{i},d_{i}\right),$$ (27)

where $\mathrm{sim}\left(l_{i},d_{i}\right)$ represents the similarity between the feature label $l_{i}$ and the disease feature $d_{i}$ in the database. The value range of $V\left(l_{i}\right)\in \left[0,1\right]$ represents the degree of accuracy of the feature label. The minimum value of $V\left(l_{i}\right)$ is 0, indicating that the feature label is not similar to any feature in the database. The maximum value of $V\left(l_{i}\right)$ is 1, indicating that the feature label is identical to a feature in the database.

The proposed similarity can be calculated based on the semantic content of the feature labels and disease features in the database. By computing the average similarity between all disease feature labels and features in the database, we can obtain an accuracy score indicating the overall accuracy of the feature labels obtained through the template search method. The manually labeled tags in the dataset tend to favor simple descriptive words. In the experiment, we simplify the sample space and feature library, making L=D; that is, the feature search library is equal to the manually labeled feature library, ensuring maximum consistency of features.

## Results

### Experimental details

In the experiment, we adopt PyTorch version 1.12 and train the model on an Nvidia A40 graphics processing unit. The training dataset consists of 2,360 images and over 20,000 manual labels. We use the stochastic gradient descent optimizer for model optimization, setting 20 epochs and a batch size of 32. For the final objective function ${\text{Object}}_{3}$, we adopt the hyperparameters α=1.2 and β=0.7. Through the application programming interfaces of OpenAI, we compare the performance of our model with cutting-edge different implementations describing generative models on several metrics, validating the superior performance of our model.

### The validation of PlanText

#### Performance comparison

To validate the comprehensive performance, we compare the proposed PlanText with adaptive attention [52], LSTNet [57], GPT-4o [44], GPT-4 [43], ClipCap [58], and Bootstrapping Language-Image Pre-training (BLIP) [59] models, and to ensure professionalism in this experiment, the textual part of our model is inputted using empty strings, and guided search templates are not present in the training set. According to Table, we can conclude 3 points.

Firstly, the unique design-space perception of the traditional excellent image description generation model LSTNet [57] allows it to perform well over regions, areas, and geographic locations of the disease. With the large models GPT-4o [44] and GPT-4 [43], the large model has high-performance scores on several feature descriptions but performs poorly on texture due to the lack of knowledge learning of pathology images.

**Table.** Comparison between the frontier model and our model on several phenotypic characteristics. Our model is better in several indicators (boldface highlighting the best outcomes).

Model	Color	Texture	Morph	Sit	Area	Addr	B@4	R-L	M
Adaptive [52]	14.35	42.41	9.74	65.84	46.82	77.74	14.32	8.65	9.17
LSTNet [57]	21.03	11.17	19.77	**83.54**	44.66	89.26	25.12	28.25	16.85
GPT-4o [44]	32.05	36.58	29.86	61.33	42.31	84.22	ND	ND	ND
GPT-4 [43]	31.19	48.62	16.51	73.39	44.95	77.98	ND	ND	ND
ClipCap [58]	47.70	57.83	38.79	82.37	60.37	85.59	32.32	34.35	32.86
BLIP [59]	**53.23**	**62.36**	25.90	72.38	44.42	57.15	52.71	**45.85**	44.34
**Ours**	32.11	48.62	**56.69**	**83.34**	**73.39**	**92.56**	**68.88**	32.26	**54.50**

B@4, BLEU-4; R-L, ROUGE-L; M, METEOR; ND, no data

In comparison, while BLIP [59] excels in color (53.23) and texture (62.36) metrics and ClipCap [57] performs well in several areas, our model obtains better results on all metrics, particularly in morph (56.69), area (73.39), and Addr (92.56). This demonstrates the effectiveness of our method in constructing a robust strong link between image phenotypes and trait descriptions, substantially enhancing the quality of generated descriptions.

Secondly, the experimental results clearly show substantial differences between various shape descriptions. Detailed research on the results generated by different models reveals that disease locations on the leaf, area, and geographical location perform better because these 3 features have larger distances, making the descriptions easier to distinguish. However, features such as color, texture, and morphology have smaller distances, making it difficult to distinguish multiple features, such as ring spots, netted spots, and random spots. ClipCap [57] and BLIP [59] utilize the Common Objects in Context (COCO) [60] dataset for model pretraining, achieving good results in color and texture. Meanwhile, on the BLEU, ROUGE, and METEOR metrics, our model performs slightly lower than ClipCap [58] and BLIP [58] on the ROUGE metric, mainly due to the attention-switching mechanism affecting the generation capability of longer sentences. However, our model shows marked advantages in BLEU and METEOR. Our 3-stage approach can somewhat overcome this issue. On the one hand, the switch-select attention mechanism enhances the embedding of descriptive text into the image descriptions. On the other hand, the model aligns the image phenotypes and traits by using a common feature space between trait labels and image samples to achieve better alignment.

Finally, traditional models face substantial challenges in plant description. One reason is the lack of large-scale image–text datasets similar to COCO [60] (over 330,000 images, 200,000 labeled images, 5 captions per image). Additionally, while large models can serve as auxiliary tools, they often cannot address specific issues in vertical domains. Therefore, our approach is necessary to obtain better image descriptions that can assist in database creation, knowledge graph construction, and the development of multimodal models for plants.

#### Text-guided ablation

To confirm the effectiveness of text bootstrapping for the model, we conduct a validation experiment.

**Fig. 9.**
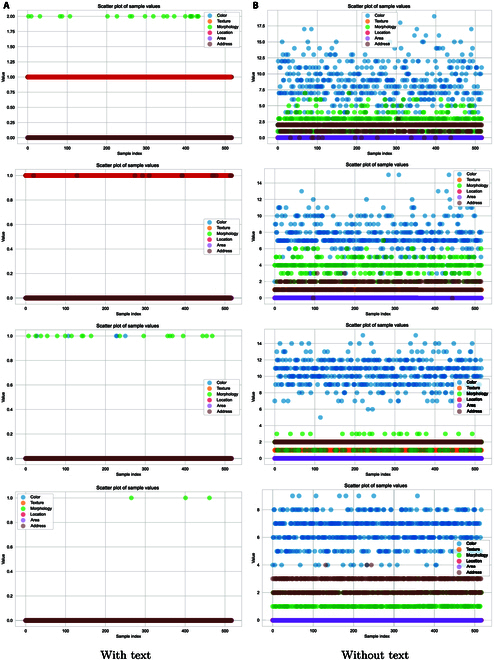
Text-guided ablation, search cost map with text-guided (A) and without text-guided (B) modeling; the horizontal coordinate indicates the ID of the images (0 to 600), and the vertical axis represents the search cost, where higher values indicate increased search difficulty. Our model through the template search tags has a search cost. (The token search number refers to the number of tokens needed to locate the first tag. A higher search cost indicates a longer time required for the search. When the model relies heavily on image phenotype features, it may yield fewer distinct outputs. Consequently, as the search cost increases, the model is more likely to produce duplicate text and may neglect extensive extraction of image phenotypes.)

We remove the text feature extractor from the model during training, employing 4 different template sentences with the template search validation method for a comparative experiment to compute the cost of searching for trait labels. We test 600 images of diseased leaves by extracting 6 phenotypic trait labels sorted by the cost required to extract them to the correct answer. The model with text guidance has a cost of 1 for disease in leaf position and shape and 0 for everything else, while the model without text guidance has a cost of 14 for color and 0 for area. As shown in Fig. 9, the model with textual guidance requires a substantially lower cost than the model without textual guidance. This effectively illustrates that text steering helps the model to understand the image content better and reflect this understanding in the generated feature markers. On the other hand, we can see that the maximum value of the cost of using the text to guide the model to search for the correct labels is 2. This shows that our model obtains the best robustness on feature labels, and further demonstrates that our model makes it possible for the model to pay better attention to the dynamic balance between the disease image and the text by switching the attention on and off. Thus, text bootstrapping plays an active role in generating feature markers and improves the model’s performance in the task of image phenotype description.

#### Visualization analysis

**Fig. 10.**
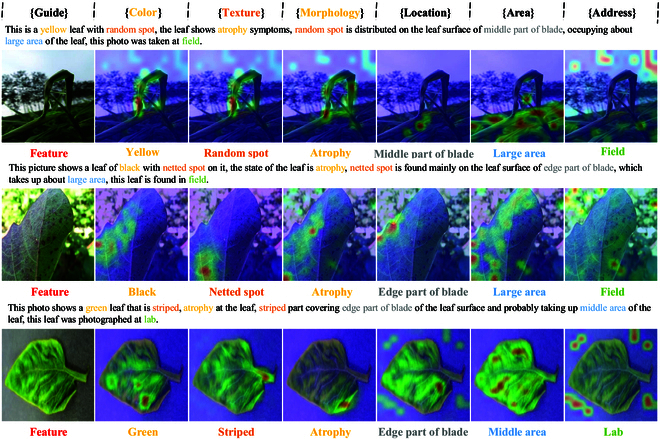
Switch attention visualization.

In order to study how attentional mechanisms are involved in recognizing lesion areas on plant leaves, we generate a heat map of the self-attention matrix from the switch attention module in the decoder part of PlanText. As shown in Fig. 10, these heat maps reflect the focus of the model’s attention on the image under the current text instruction. First, comparing the original image with the attention heat maps of the instructional commands, we clearly observe marked hotspots in specific regions of the leaf image for different commands. The reason is that our model is able to effectively focus on critical regions during image processing, thus enhancing its ability to recognize plant diseases, proving the effectiveness of the switch attention mechanism we employed. Second, comparing the heat maps of different trait descriptors, our model’s image-switch attention effectively focuses on specific regions in each trait label extraction task. This demonstrates that our model performs well in the disease recognition task, with the switch attention mechanism playing a key role in ensuring the accuracy and robustness of the model. All in all, these observations further strengthen our understanding of our model’s performance and provide strong support for the switch attention mechanism we employed.

### Application generalizability

#### Trait analysis of different diseases in apple

**Fig. 11.**
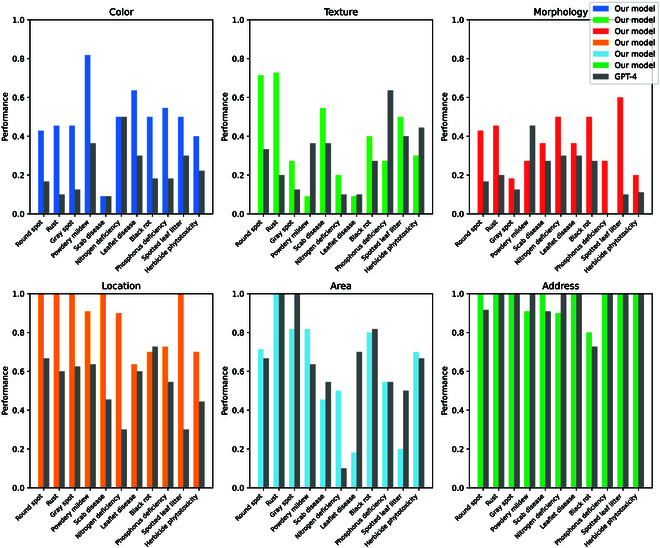
Our model’s results in assessing various apple diseases.

To validate the applicability of our model to a single plant, as shown in Fig. 11, we evaluate it on various apple diseases, including round spot, rust, gray spot, powdery mildew, scab disease, nitrogen deficiency, leaflet disease, black rot, phosphorus deficiency, spotted leaf litter, and herbicide phytotoxicity. During this evaluation, we compare our model’s performance to that of the highly regarded GPT-4 [43].

Across various apple diseases, our model demonstrates clear advantages. First, in terms of trait attributes, our model only slightly lags behind GPT-4 [43] for some specific disease traits, demonstrating its high information dimension and clear presentation of disease distribution on different apple leaves. However, when considering multiple apple diseases, our model outperforms GPT-4 [43] in all cases, thanks to its image-switch attention mechanism, which allows for more accurate differentiation between diseases. Additionally, during testing, our model achieves a recognition speed of 12 frames per second on an Nvidia A10 graphics card, indicating lower cost and higher recognition speed, providing comprehensive, fast, and practical support for research and management decisions.

#### Trait analysis of powdery mildew disease in different plants

**Fig. 12.**
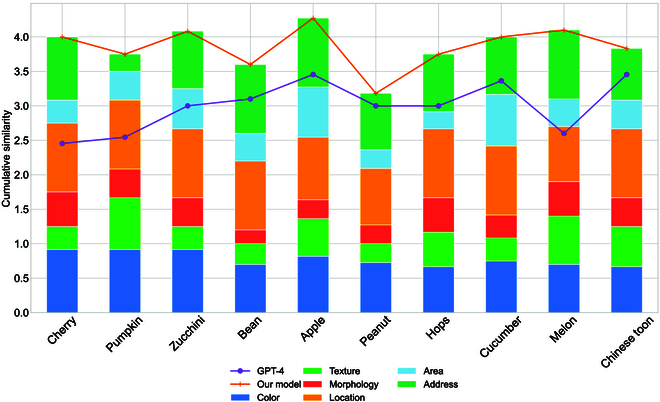
Our model’s results on powdery mildew disease across different plants.

To assess the robustness of our model in identifying individual diseases, we evaluate the powdery mildew disease on various plants such as cherry, pumpkin, zucchini, bean, apple, peanut, hops, cucumber, melon, and Chinese toon, as shown in Fig. 12. In the powdery mildew disease of different plants, the stacked bar chart accurately depicts the description accuracy of our model’s 6 features, while the line chart shows the cumulative accuracy of our model and GPT-4 [43] (to strengthen the experiment, we use strict accuracy instead of similarity, meaning the feature label similarity can only be 0 or 1, with the cumulative accuracy ranging from 0 to 6).

First, in terms of trait attributes, the stacked bar graphs show that although there is still room for improvement in recognizing morphological and area traits, the overall performance of our model is substantially better than that of GPT-4 [43], especially in terms of cumulative descriptive accuracy for various plant diseases. Second, comparing the accuracy of cumulative trait descriptions, our results outperformed GPT-4 [43] in terms of powdery mildew per plant. These 2 results effectively demonstrate that our model exhibits high accuracy in describing powdery mildew on different plants. Finally, when considering multiple apple diseases, our model outperforms GPT-4 [43] in all cases. This phenomenon demonstrates the robustness of the model in terms of trait and phenotype alignment across plants, while also demonstrating a variety of different shortcomings.

### Defect analysis

#### Defect display of PlanText

To compare the effectiveness of different methods in describing the same disease pictures, we assess their performance in perceiving different types of attributes from a qualitative perspective. We selected several cutting-edge models to describe disease pictures, including adaptive attention [52], LSTNet [57], and GPT-4 [43] models and our PlanText model. We perform a qualitative analysis of the descriptions generated by each method, focusing on attributes such as color, region texture, and pathology morphology in the descriptions.

**Fig. 13.**
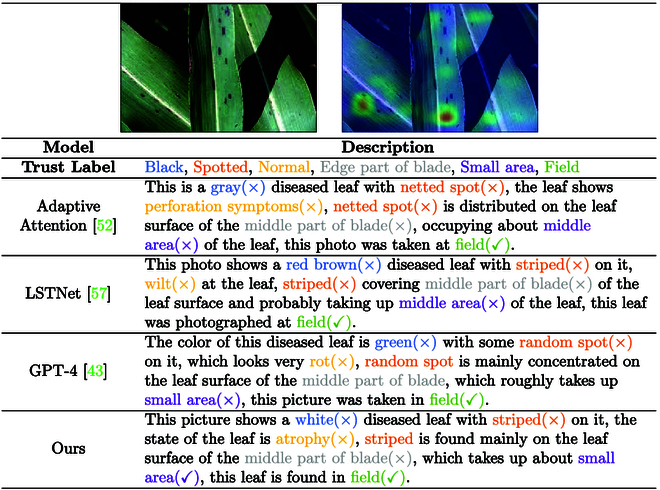
Comprehensive model evaluations on leaf disease detection.

Based on the results in Fig. 13, we find that the methods perform relatively poorly in 3 attributes: color, region texture, and pathological morphology. This finding may be attributed to the model’s inadequate understanding of the subtle features of the plant in the image. First, in terms of color, there may be similarities with the background or variations in color due to lighting conditions, which makes it difficult for the model to accurately distinguish the true color of the corn leaf. Second, regarding area texture, leaf surfaces may exhibit complex texture structures and be affected by occlusion from other objects or interference from complex backgrounds, making it difficult for models to accurately capture texture details. In addition, the pathological morphology of leaves may be affected by disease, natural growth, or other factors that result in morphological changes or abnormalities, making it difficult for models to accurately characterize the true morphology of leaves. As a result, these factors combined affect the model’s ability to accurately identify features such as color, regional texture, and pathological morphology, resulting in lower scores.

In conclusion, our experiment shows that the existing methods have deficiencies in recognizing the color, regional texture, and pathological morphology of plant leaves in images.

#### Data defect

The accuracy and consistency of data labeling are critical to model performance. However, in practice, due to the subjectivity of the annotators and the diversity of descriptions, data annotation often suffers from inconsistency, which affects the training effect and prediction accuracy of the model. In order to evaluate the performance of the models under different labeling conditions, we select 4 different models for the experiments, including adaptive attention [52], LSTNet [57], GPT-4 [43], and our self-developed model. Each model is labeled and tested in 6 aspects: color, texture, morphology, location, area, and address, respectively. Additionally, we introduce a trust label as a reference to evaluate the labeling accuracy and consistency of the models.

**Fig. 14.**
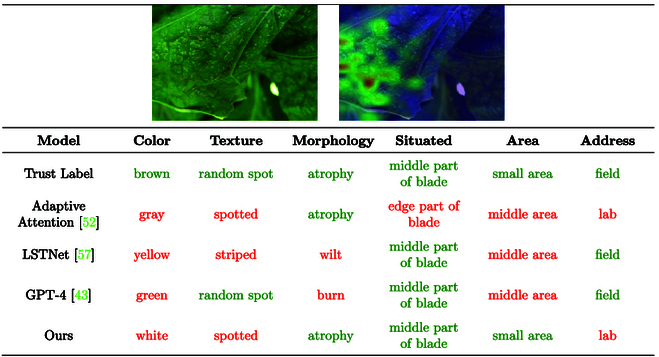
Detailed analysis of model performances.

Fig. 14 shows the labeling results of each model for different features. Specifically, the adaptive attention [52] model blurrily distinguishes between gray and yellow in color annotation. The LSTNet [57] model confuses stripes and wilts in texture annotation. The GPT-4 [43] model makes an error in annotating random patches in recognizing burned texture, and our self-research model correctly identifies white patches in color labeling. Through comparative analysis, we find that inconsistencies and vague descriptions in manual annotation lead to mismatches between phenotypes and traits. Specifically, during manual annotation, humans may favor visual impressions of color and texture, potentially overlooking widespread disease coloration. In contrast, the model relies solely on historical images for judgment, resulting in discrepancies between model predictions and manual annotation results. To address the problem of data labeling, we explore active learning methods to optimize the model training process by selectively labeling difficult and critical samples through continuous interaction with the model. At the same time, an incremental labeling strategy is used to gradually improve and refine the labeled data to adapt to the needs of the model and the changes of new data.

## Discussion

### Model development and performance

In this study, we develop a phenotype and trait description model that gradually masks bootstrap images to align plant disease text. To synchronize the gaps in the plant disease multimodal training dataset, we collect 21,000 disease images for annotation to construct a large and rich dataset. Ultimately, we use this dataset to train our model to effectively characterize the features and conditions of plant disease images based on the given images and templates guiding description generation.

Experimental results show that our model is well trained using global and heterogeneous features to switch attention and dynamically balance disease phenotypic trait descriptions and textual context. The phenotypic trait descriptions generated by the final model are rich in content and are able to capture various features of plant leaves effectively.

Additionally, we utilize a phenotypic trait label extraction method to extract labels using the existing image caption model. Leveraging the retrieval capability of the large-scale language model, we offer reasonable suggestions and implement plans for plant disease control, providing valuable support for agricultural production.

### Data integration in intelligent agricultural systems

Our approach combines image description models, such as our model or others, with phenotypic trait label extraction methods to generate plant disease image labels accompanied by detailed textual descriptions. After manual screening and validation, this process enables the creation of a comprehensive database with substantial potential applications in agriculture.

Firstly, despite the wealth of expert-labeled data, relying solely on manual labor for database construction is impractical due to the time and expertise required for manual diagnostics. Therefore, combining expert annotation with image description models, and label extraction generation, followed by manual inspection, effectively reduces time and labor costs.

Secondly, the database includes labels for various disease traits, facilitating the development of advanced machine-learning models for automated plant disease diagnosis. This capability aids agricultural practitioners in detecting and treating diseases early, thereby enhancing crop yield and quality.

Lastly, the labeled textual data derived from the image outputs in the database serve as inputs for developing smart agricultural systems. Real-time monitoring of plant health optimizes agricultural production processes, minimizing resource wastage. Industry professionals can devise more scientific and effective strategies for disease control, thereby reducing losses.

This research represents a valuable resource for plant health and agricultural management, providing robust support for future technological innovations and sustainable agricultural development.

### Future directions

**Fig. 15.**
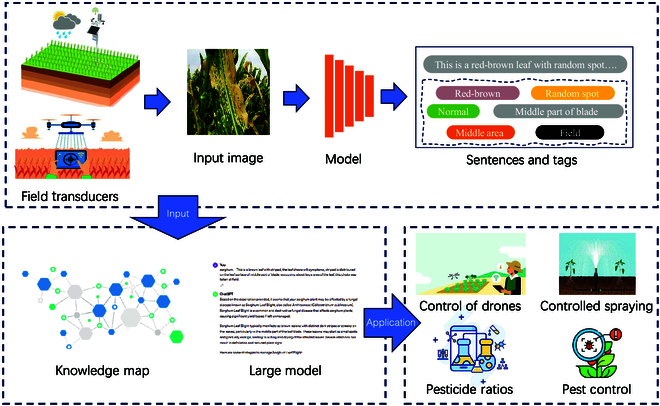
Our model’s results in apple disease assessment.

Future research directions are included but not limited to the following aspects (see Fig. 15):

Firstly, we can further improve the training algorithms and architectures of the model to enhance the accuracy and diversity of generated descriptions. For example, exploring the application of image segmentation techniques can partition plant leaf images into finer-grained regions, enabling the model to describe the characteristics of each region more accurately.

Secondly, we can further leverage the retrieval capabilities of large language models to extract more effective information about plant disease prevention and control from massive agricultural literature and data. By integrating the model with real-time data streams, we can realize a real-time intelligent decision support system, providing timely advice and guidance to farmers and agricultural experts.

Additionally, we can consider transforming our model into electrical signals to drive machines for real-time monitoring and control of plant diseases. By integrating with agricultural smart equipment such as drones or robots, automated agricultural production management can be achieved, improving production efficiency and crop quality.

Lastly, we can explore the potential applications of the model in other fields such as forestry and horticulture. By further optimizing the performance and application of the model, we provide more comprehensive and effective solutions for a wider range of agricultural and botanical fields, promoting the modernization and intelligence of agricultural production.

### Applicability of PlanText

The PlanText framework provides a structured approach for aligning image data with textual descriptions, which is crucial for effective communication in agricultural contexts. To enhance its applicability, we recognize that certain methods within the framework may impose limitations. Therefore, we propose several adaptability requirements to ensure that the framework can meet the diverse needs of practical applications.

Firstly, the framework must accommodate a variety of crops and plant diseases. Different crops exhibit distinct phenotypic traits and may require tailored approaches to description generation. Future iterations of the framework could incorporate modular components that allow users to customize the model based on the specific crops and diseases being addressed. This modularity would enable practitioners to select relevant features and characteristics pertinent to their agricultural context. Secondly, the adaptability of the PlanText framework should consider varying environmental conditions. Plant health is influenced by factors such as climate, soil type, and water availability. To make the framework more versatile, we can integrate environmental data inputs that inform the model about the conditions under which the plants are growing. By doing so, the model can provide more context-aware descriptions and recommendations. Additionally, user feedback mechanisms should be incorporated into the framework. Engaging end users, such as farmers and agricultural experts, in the iterative refinement of the model would allow the framework to evolve based on real-world challenges and needs. This participatory approach ensures that the generated descriptions remain relevant and practical. Lastly, the framework should support integration with other agricultural technologies and data sources. By allowing seamless interoperability with existing agricultural management systems, sensor data, and other AI-driven tools, the PlanText framework can enhance its utility. This integration will provide a holistic view of plant health and facilitate real-time decision-making.

In summary, while the PlanText framework offers a solid foundation for our model, enhancing its adaptability will substantially expand its applicability in various agricultural settings. By addressing these adaptability requirements, we can better align the framework with the practical needs of users in the field, ultimately leading to more effective plant disease management solutions.

### Conclusion

This study presents a model for describing plant diseases using a dataset of 21,000 annotated images, enhancing feature characterization. It integrates image descriptions and phenotypic labels to create a comprehensive database, facilitating automated disease diagnosis and early intervention. Future work will focus on improving model accuracy, integrating real-time data for decision support, and expanding applications to various agricultural fields. The PlanText framework aims to align image data with textual descriptions, requiring adaptability for different crops, environmental conditions, user feedback, and integration with existing technologies for effective plant disease management.

## Data Availability

We release our data at https://plantext.samlab.cn.
